# Novel early vertical ridge augmentation technique

**DOI:** 10.1016/j.jds.2024.10.025

**Published:** 2024-11-06

**Authors:** Jerry Chin-Yi Lin, Shaima O. Bahammam, David M. Kim, Wei-Jen Chang

**Affiliations:** a School of Dentistry, College of Oral Medicine, Taipei Medical University, Taipei, Taiwan; b Department of Oral Medicine, Infection and Immunity, Harvard School of Dental Medicine, Boston, MA, USA; c Dental Department, Shuang-Ho Hospital, Taipei Medical University, New Taipei City, Taiwan

**Keywords:** Vertical ridge augmentation, Early bone augmentation, Post-extraction site management, Tenting screws, Allografts, Xenografts

## Abstract

**Background/purpose:**

Multiple augmentation protocols are documented in the literature to rebuild the deficient alveolar ridge after tooth extraction; however, achieving adequate vertical augmentation remains the most challenging goal. This study demonstrated a novel surgical technique of early vertical ridge augmentation for post-dental extraction. This technique offers several biological and technical advantages regarding the timing of the procedure and its relative simplicity compared to other complex techniques.

**Materials and methods:**

This retrospective study consisted of 50 extraction sites from 44 participants who had received early vertical ridge augmentation (VRA) procedures (6–16 weeks post-extraction in either the maxilla or the mandible). The procedures were carried out using titanium tenting screws, freeze-dried bone allografts (FDBA), and xenografts (bovine). Pre- and post-operative cone-beam computed tomography (CBCT) images were taken for all participants 6–9 months after the augmentation surgery to measure the vertical bone gained.

**Results:**

A total of 44 patients were treated with early VRA 6–16 weeks post-dental extraction. The total mean vertical bone gain measured after 6–9 months of augmentation for all cases was 4.64 ± 1.76 mm with no complications encountered. About 80 % of augmented sites met or exceeded the expected vertical bone gain (EVBG). Delaying vertical ridge augmentation until after eight weeks post-extraction, particularly beyond twelve weeks, results in higher rates of EVBG and more consistent average bone gain.

**Conclusion:**

This study indicates that VRA can be achieved predictably by utilizing the early VRA technique, which is relatively straightforward and is associated with a minimal complication.

## Introduction

Published literature supports performing alveolar ridge preservation (ARP) to reduce ridge alterations and atrophy after tooth extraction.^1^ ARP demonstrated horizontal bone resorption prevention, followed by vertical mid-buccal and vertical mid-lingual bone changes and volumetric soft tissue changes compared to spontaneous socket healing.^2^ This reduces the need for additional complex bone augmentation procedures later on.^2^ However, ARP may result in complications such as difficulties in wound closure, post-operative membrane exposure, delay in bone maturation, etc. In addition, ARP may not eliminate the need for additional bone augmentation at the time of implant placement when compared with natural socket healing.^3^

Multiple late bone augmentation protocols have been documented in the literature to rebuild the deficient alveolar ridge before implant placement, including bone blocks, distraction osteogenesis, segmental osteogenesis, and guided bone regeneration (GBR).^4^ Different surgical protocols and multiple materials have been developed, including resorbable and non-resorbable membranes utilized alone or in conjunction with bone grafting materials, titanium pins, meshes, and tenting screws, and their effective outcomes have been reported.^4^, ^5^, ^6^, ^7^, ^8^ However, achieving adequate vertical ridge augmentation (VRA) with GBR remains challenging for most clinicians.^9^

The first human histologic evidence for vertical augmentation of an atrophic ridge with a non-resorbable expanded polytetrafluoroethylene (e-PTFE) titanium-reinforced membrane without the use of bone graft around dental implants was reported by Simion et al., in 1994.^10^ Histologic measurement showed a 3–4 mm gain in bone height around the protruded implants. Tinti et al., 1996 reported an average vertical bone gain of 4.95 mm with the addition of autogenous bone to the e-PTFE titanium-reinforced membrane.^11^ Similar to the autogenous bone, adding demineralized freeze-dried allograft (DFDBA) with the e-PTFE titanium-reinforced membrane demonstrated beneficial clinical and histologic evidence on VRA.^12^ Other studies evaluated the efficacy of deproteinized bovine bone mineral (DBBM) used alone or mixed with autogenous bone associated with e-PTFE titanium-reinforced membrane.^13^^,^^14^ Clinical and histologic findings showed successful and predictable vertical reconstruction of atrophic ridges using DBBM and e-PTFE titanium-reinforced membrane. Urban et al.^15^ published a prospective case series evaluating a titanium-reinforced d-PTFE membrane combined with a mixture of an organic bovine bone-derived mineral (ABBM) and autogenous bone particles for VRA. Results exhibited successful vertical bone formation in 20 deficient sites with an average of 5.45 mm bone gain. In a recent systematic review by Urban et al., in 2019, where the effect of various vertical augmentation protocols reported in 36 studies was compared, non-resorbable PTFE membranes showed higher bone gain than resorbable membranes (4.42 versus 3.51 mm).^4^ Although multiple studies demonstrated predictable alveolar bone regeneration, vertical augmentation procedures are still technique-sensitive and associated with an increasing risk of complications and morbidity for the patients.^4^^,^^16^ Therefore, continuous efforts have been made to develop predictable methods and protocols for VRA.

In situations where there are substantial alveolar soft and hard tissue deficiencies after either a single tooth or multiple adjacent teeth extractions, soft tissue management can be quite challenging during ARP, such as a single first molar extraction with the presence of the second molar. A new augmentation concept that tackles the challenges in ARP and other late bone augmentation techniques is proposed in the present study, referred to as the “Early Bone Augmentation Protocol.” Soft tissue is allowed to mature for 6–16 weeks following extractions. Then, vertical and horizontal ridge augmentation with bone grafts, including allograft, xenograft, or both, and resorbable membrane via GBR techniques are implemented. During this healing time, several biological events favor the clinical results. This concept is based on utilizing the healing potential at around an 8-week period where the bone regeneration is at its peak with maximum osteogenic activity and the highest density of vascular structures, observed as the proliferation of cellular and connective tissue elements and osteoblast laying down osteoid around immature bone islands.^17^, ^18^, ^19^, ^20^, ^21^ By that time, the soft tissue had become thicker and had enhanced vascularity compared to the time of extraction, which offered easier soft tissue management and tension-free primary closure.^19^^,^^20^^,^^22^

The proposed augmentation technique offers several biological and technical advantages. It is a straightforward technique using commonly available biomaterials, including particulate bone grafts (allograft or xenograft), resorbable collagen membrane (eliminating the need for a second surgery to remove the membrane), and tenting screw (maintaining the space and preventing the collapse of the membrane). Non-resorbable d-PTFE membranes may be used in special situations for enhanced space maintenance. Moreover, it allows acute or chronic infections to resolve, offering a lower risk of infection at the augmentation site. All these changes within this early healing phase simplify the surgical technique and reduce postoperative complications.

In this retrospective study, we provided detailed information and demonstrated a step-by-step surgical procedure of the novel early bone augmentation technique in 50 sites from 44 patients. The outcomes were analyzed by evaluating the clinical and radiographic parameters for vertical bone gain.

## Materials and methods

### Study design and population

This retrospective study included participants who had received early VRA procedures 6–16 weeks post-extraction in either the maxilla or the mandible. IRB was approved by the Joint Institutional Review Board at Taipei Medical University (Approval Number: N202405024). Participants presented with good physical health and oral hygiene, including the pre-operative cone-beam computed tomography (CBCT) before GBR. Exclusion criteria included diabetic patients, patients with uncontrolled systemic diseases, and patients on drugs that may affect bone metabolism. One experienced periodontist (JL) treated all cases from May 2016 to Dec 2023.

### Surgical protocol

**Figure 1:**
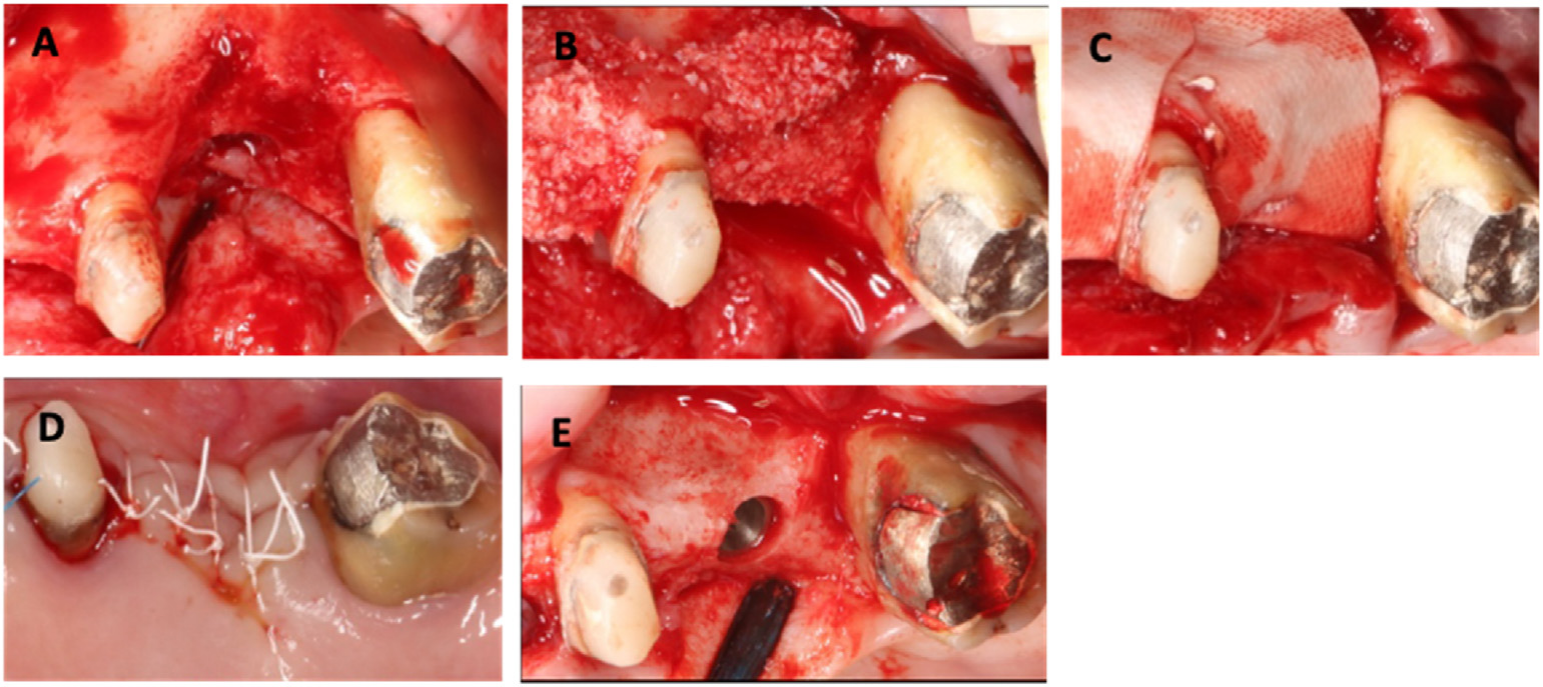
A clinical case showing the steps of the early bone augmentation procedure without the use of a tenting screw. (A) Alveolar defect in the sites of extracted teeth #26. (B) The sites were augmented with a mixture of xenograft and allograft. (C) The bone graft particles were covered with a cross-linked collagen membrane. (D) The site was sutured with 4-0 dPTFE sutures, 5-0 polypropylene and 5-0 Chromic gut. (E) The augmentation sites were accessed 21 months later for implant placement, showing the amount of bone gained vertically.

**Figure 2:**
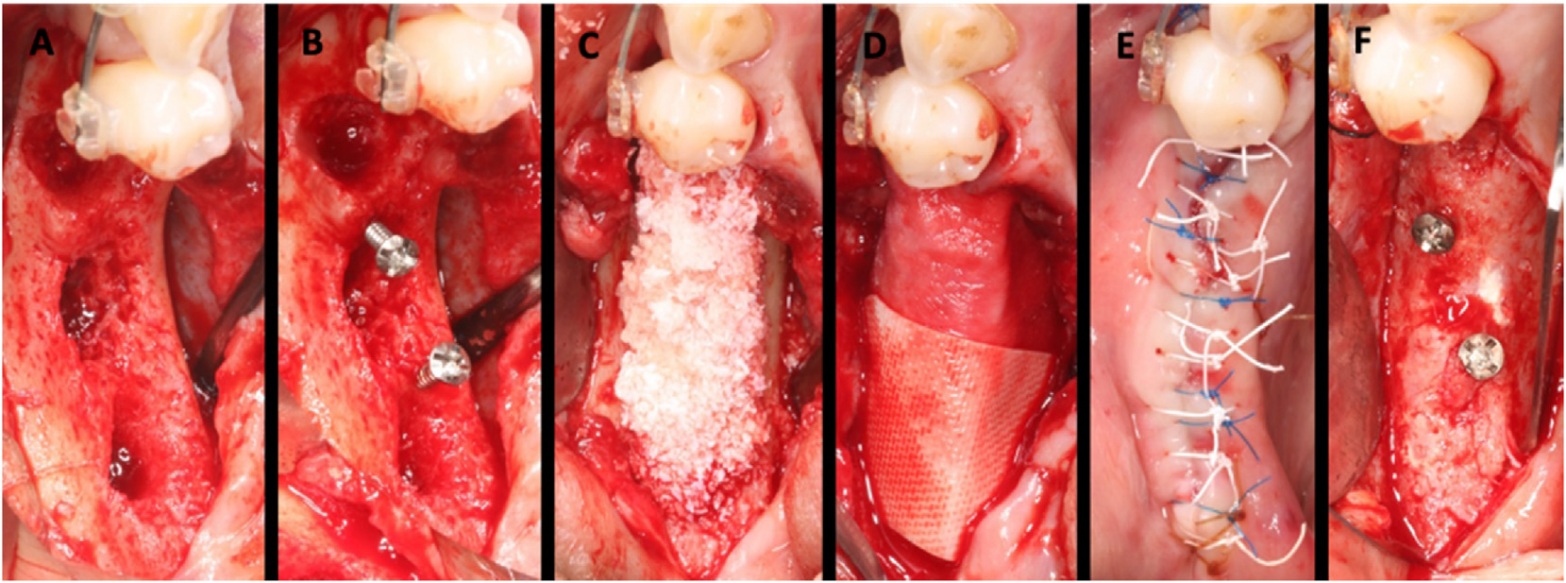
A clinical case showing the steps of the early bone augmentation procedure with the use of tenting screws. (A) Alveolar defect in the sites of extracted teeth #35–37. (B) Two tenting screws were placed in the defect to maintain the space. (C) The sites were augmented with a mixture of xenograft and allograft. (D) The bone graft particles were covered with a cross-linked collagen membrane. (E) The site was sutured with 4-0 dPTFE suture and 5-0 polypropylene suture. (F) The augmentation sites accessed 5 months later for implant placement demonstrated a significant amount of horizontal and vertical bone gain.

Teeth that were indicated for removal were extracted under local anesthesia. The extraction sites were allowed to heal for 6–16 weeks for complete soft tissue healing. Before the augmentation procedure, the oral cavity was rinsed with 0.12 % chlorhexidine solution for 30 s. After administration of local anesthesia with 2 % Lidocaine 1:100,000 epinephrine, a bucco-crestal or mid-crestal incisions were made within the keratinized tissue on the edentulous alveolar ridge, and intrasulcular incisions were extended at least one tooth mesially and distally. Vertical incisions were made mesially and distally to the defects. Full-thickness mucoperiosteal flaps were reflected buccally and lingually. All soft tissue remnants in the premature sockets were removed. Intra-marrow penetration was made with a carbide round bur under copious irrigation. Titanium tenting screws with 1.8 mm in diameter and 3 mm in head diameter (WY Biomedical Co., Taipei, Taiwan) was inserted when indicated at least 3 mm into the bone within the defect with the screw head flushing the future bone level. The defect was grafted with bone substitutes with different combinations, including mineralized allograft (DIZG, gemeinnützige GmbH, Berlin, Germany) alone, xenograft alone (The Graft, Purgo Biologics, Seongnam, South Korea), or allograft with a thin layer of xenograft (DBBM - Geistlich Bio-Oss®, Geistlich Pharma AG, Walhusen, Switzerland) on top of the allograft. The bone graft was then covered with collagen membranes (Ossix Plus, Datum Dental, Lod, Israel). Tension-free closure was achieved with buccal periosteal release or in combination with lingual release for the posterior mandible. The flaps were then adapted and sutured with 4-0 dPTFE for the horizontal mattress sutures and with 5-0 Polypropylene/5-0 Nylon/5-0 chromic gut for simple loop interrupted sutures (Figure 1, Figure 2).

### CBCT bone measurement

**Figure 3:**
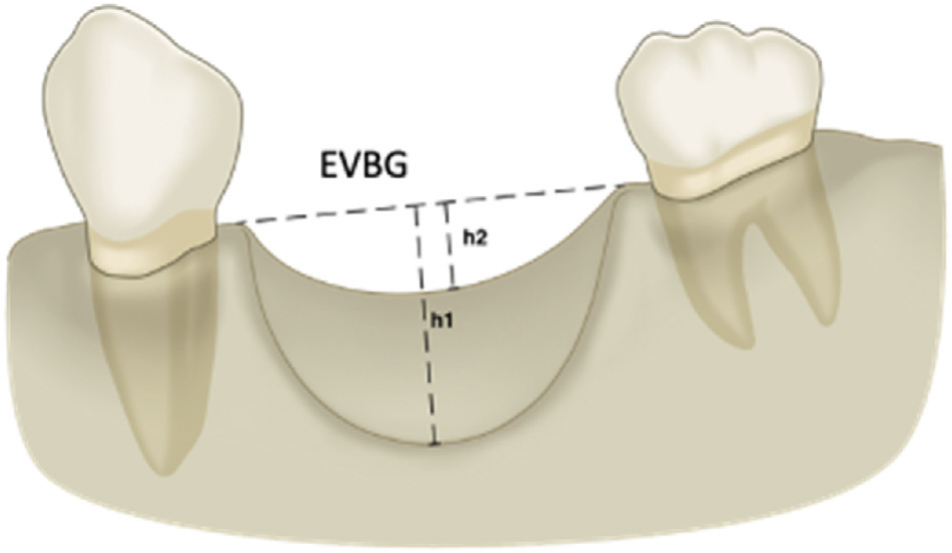
The expected vertical bone gain was measured by drawing a line between the existing residual mesial and distal bone peaks adjacent to the defect. EVBG: expected vertical bone gain, h1: indicates the maximal defect depth, h2: indicate true vertical gain.

Post-operative CBCT images were taken for all participants 6–9 months after the augmentation surgery to calculate the vertical bone gained. Pre-operative and post-operative CBCT images were superimposed, and the amount of vertical bone gain was measured using Simplant (Dentsply Sirona, PA, USA) software. A line was drawn between the existing residual mesial and distal bone peaks adjacent to the defect to calculate the expected vertical bone gain (EVBG) (Fig. 3). Then, the difference between the true vertical bone gain (TVBG) and the EVBG was calculated (h2 indicated true vertical gain while h1 indicated the maximal defect depth). A positive value was assigned in cases where bone gain exceeded the EVBG, and a negative value in cases where the TVBG was less than the EVBG. One calibrated examiner performed all of the measurements.

### Statistical analysis

Descriptive data are presented as means ± standard deviations (SDs). Differences in bone level gain among the groups were examined using one-way ANOVA. Pearson correlation tests were performed to analyze the relationships between time and EVBG, as well as time and vertical bone gain. Statistical analyses were performed using Prism (v10; GraphPad Software Inc., Boston, MA, USA). Values of *P* < 0.05 were considered statistically significant.

## Results

A total of 50 sites in 44 patients were treated with early augmentation 6–16 weeks post extraction. The mean patient age was 55 years (range: 38–77 years); two-thirds were women (n = 30), and one-third were men (n = 14). Most of the augmented sites were single missing teeth (n = 30), while the remaining 20 sites were multiple adjacent missing teeth. Cases were distributed between the maxillary and the mandibular teeth (Table 1).

**Table 1:** Distribution of cases between the maxillary and the mandibular teeth.

		Incisors	Canine	Premolar	Molar	Total	
Maxilla	Single tooth	4	1	1	7	13	27
	Multiple teeth	4	1	1	8	14	
Mandible	Single tooth	3	1	1	12	17	23
	Multiple teeth	1	–	–	5	6	
Total		12	3	3	32		50

**Figure 4:**
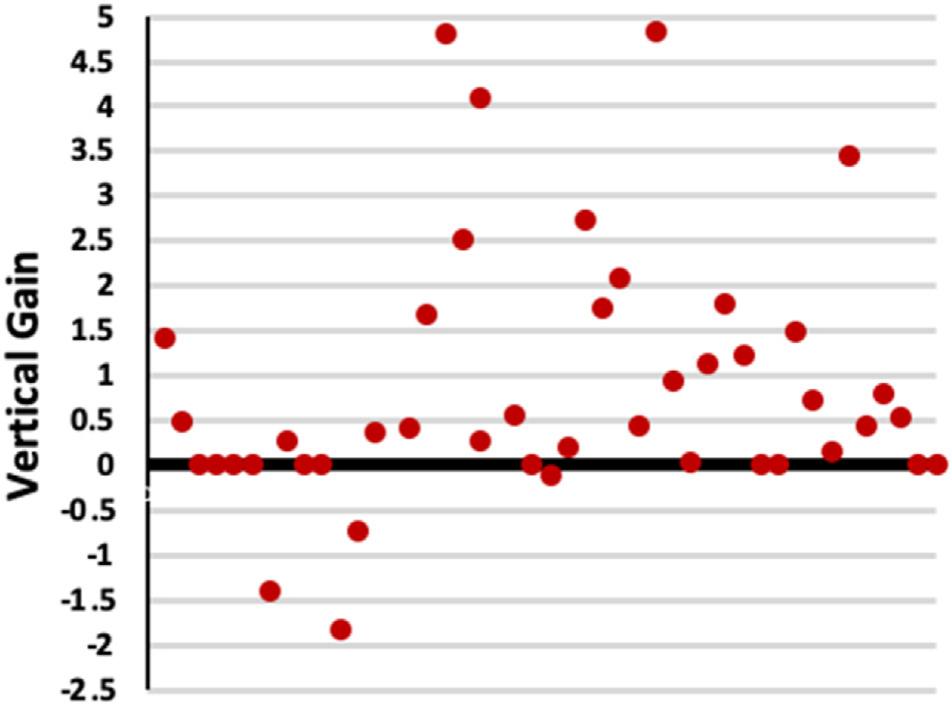
True vertical gain of all sites relative to the expected vertical bone gain (EVBG, Y = 0). Sites that exceeded the EVBG have a value above 0.

All patients experienced uneventful healing with no post-operative complications encountered. The total mean true vertical bone gain measured after 6–9 months of augmentation for all cases was 4.64 ± 1.67 mm. About 80 % of augmented sites met or exceeded the expected vertical bone gain by +1.45 ± 1.47 mm, whereas the rest of the cases fell slightly shorter by −0.96 ± 0.78 mm (Table 2) (Fig. 4).

**Table 2:** Measurement of the total mean true vertical bone gain after 6–9 months of augmentation for all cases.

	≥ EVBG	< EVBG
n	40	10
%	80	20
Mean ± SD (mm)	+1.45 ± 1.47	−0.96 ± 0.78

EVBG: expected vertical bone gain.

To evaluate the relationship between healing time after extraction and the amount of true vertical height obtained after augmentation, the data were divided into before-8-week and after-8-week groups. Subsequently, the after-8-weeks group was further subdivided into 8-12-week and after-12-week groups. The before-12-week data was also analyzed for further comparison. The percentage of sites exceeding the EVBG and the average bone gain were analyzed and presented in Table 3.

**Table 3:** The percentage of sites exceeding the expected vertical bone gain and the average bone gain.

	Number of sites	% Sites Exceeding EVBG	Average bone gain
Before 8 weeks	16	62.5	4.63 ± 2.03
After 8 weeks	34	88.23	4.64 ± 1.52
8–12 weeks	23	86.95	4.62 ± 1.42
Before 12 weeks	39	76.92	4.62 ± 1.67
After 12 weeks	11	90.90	4.69 ± 1.78

EVBG: expected vertical bone gain.

**Figure 5:**
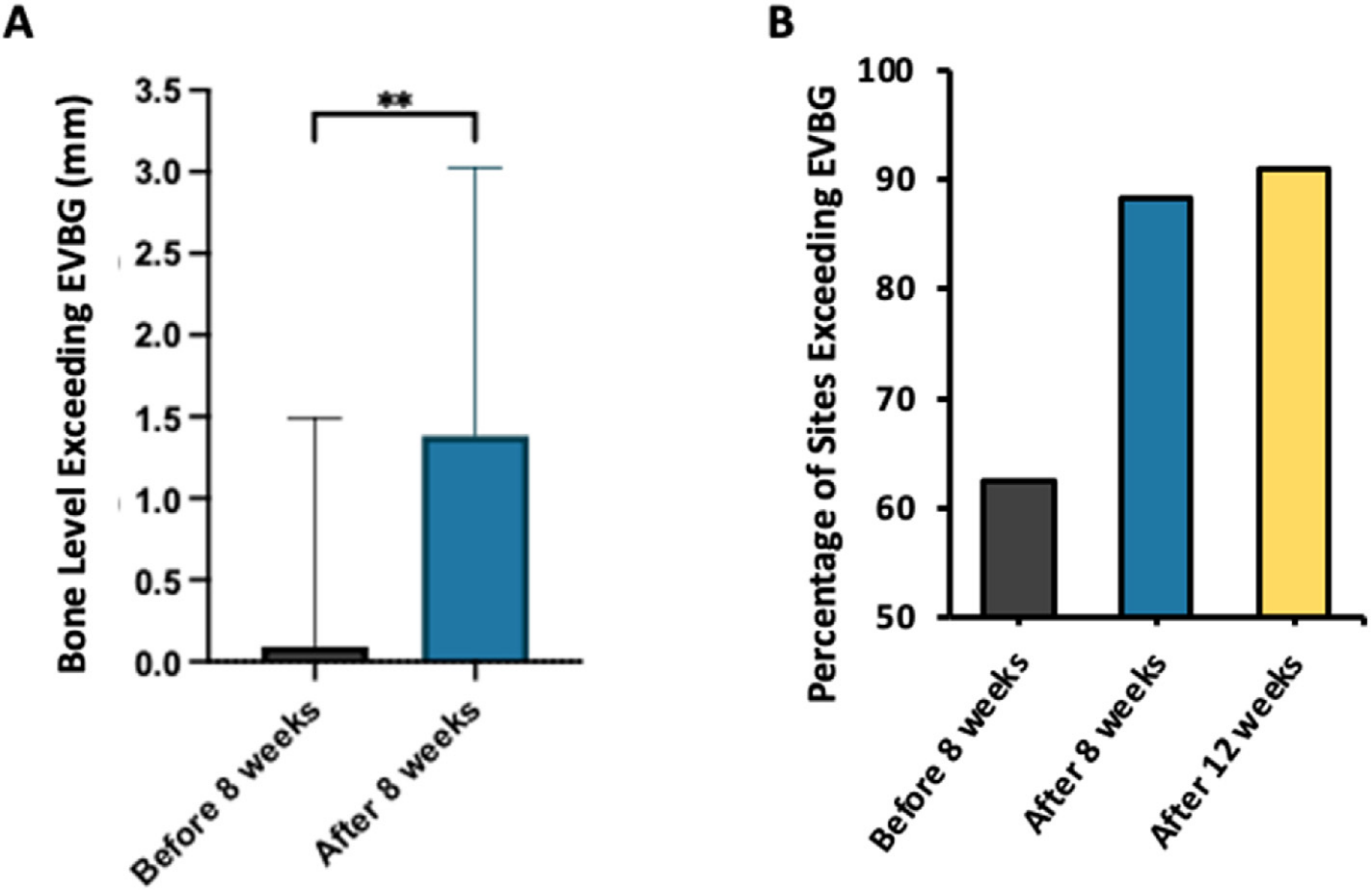
(A) A bar graph demonstrating the bone level exceeding the expected vertical bone gain (EVBG) in the before and after 8-week groups (one-way ANOVA, *P* = 0.03). (B) A bar graph showing the percentage of sites that exceeding the EVBG in before 8-week, after 8-week, and after 12-week groups. EVBG: expected vertical bone gain.

The bone level gained and the extent to which it exceeded the EVBG was calculated and compared across all the groups. Notably, only the group assessed after 8 weeks exhibited a significant increase in average bone level gain (*P =* 0.03) compared to the group assessed before 8 weeks, with a higher percentage of sites exceeding the EVBG (Fig. 5). Specifically, the after-8-week group, 88.23 % of the sites exceeded EVBG, with an average bone gain of 4.64 ± 1.52 mm, whereas the before-8-week, 62.5 % of the sites exceeded EVBG, with an average bone gain of 4.63 ± 2.03 mm.

**Figure 6:**
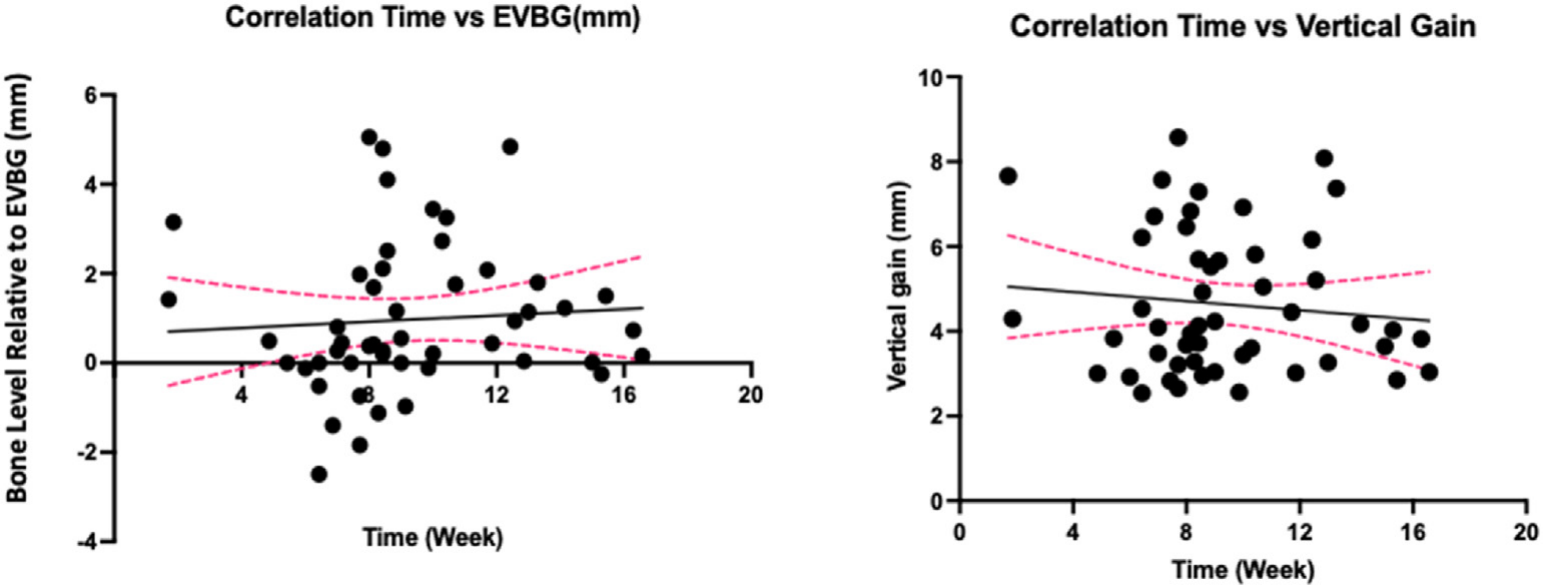
(A) A plot graph illustrating the correlation between time and the bone level gained in all sites relative to the expected vertical bone gain (EVBG). There is a positive correlation (r = 0.2377) between time and the amount of bone level gained relative to the EVBG. (B) A plot graph illustrating the correlation between time and average vertical bone gain in all sites. It shows a slightly negative correlation between time and the average vertical bone gained. EVBG: expected vertical bone gain.

Within the after-8-week group, 86.95 % of the sites in the 8-12-week subgroup exceeded EVBG, with an average bone gain of 4.62 ± 1.42 mm. In comparison, 76.92 % of the sites in the before-12-week subgroup exceeded EVBG, with an average bone gain of 4.62 ± 1.67 mm. Notably, 90.90 % of the sites in the after-12-week subgroup exceeded EVBG, demonstrating the highest EVBG, with an average bone gain of 4.69 ± 1.78 mm. Overall, the data reveal a pattern where the incidence of bone gain exceeding the EVBG increases over time (Fig. 6).

## Discussion

Vertical ridge augmentation is a great challenge for clinicians due to its complexity in surgical techniques and unpredictable treatment outcomes. Nonetheless, guided bone augmentation techniques have been demonstrated to be effective in achieving vertical bone gain with different techniques and materials, such as titanium-reinforced PTFE membranes in conjunction with blood clots,^10^ autografts,^11^ allografts,^12^ xenografts,^13^ or their mixtures.^14^ However, certain complications related to the PTFE membranes for vertical ridge augmentation, such as early membrane exposure, infection, abscess, and surgical complications,^23^ may occur up to 45.5 % of the time.^24^ On the contrary, resorbable membrane offers better soft tissue healing and self-limiting infection,^25^ reducing surgical difficulties and post-operative complications. Although limited evidence indicated the gain of VRA with resorbable membrane, certain promising results could be achieved.^26^ Data from a systemic review and meta-analysis for vertical ridge augmentation by Urban et al.^4^ in 2019 indicated a mean vertical bone gain of 4.16 mm for all treatment modalities and 4.18 mm for GBR, in which 4.42 mm for the non-resorbable membrane and 3.51 mm for the resorbable membrane group. The present study achieved a mean true vertical ridge augmentation of 4.64 mm with a maximum bone gain of 8.57 mm for the true vertical defects where both buccal and lingual walls of bone were lost. The results of this study presented comparable vertical bone gain to the non-resorbable membrane GBR and superior to the resorbable membrane GBR.

Surgical complications were not uncommon for VRA. The overall complication rate of 16.9 % was wide-ranging among different approaches.^4^ The cases included in the present study did not encounter any serious post-operative complications except one with an incision line opening at the time of suture removal, which was resolved with spontaneous healing and left no adverse effects on the results. Moreover, the result of the VRA still exceeded the expected bone-to-bone level.

Based on the literature,^17^^,^^20^ the bone regeneration was observed to peak around 8 weeks after extraction. The relationship between time and healing potential regarding clinical relevance has not yet been established and examined. Based on the present study, within the 16-week interval among 50 sites, 80 % of the time, the VRA exceeded the anticipating level (EVBG). The average vertical bone gain was 4.64 mm. Using 8 weeks post-extraction as a cut-off demonstrated that better results were obtained from sites with vertical bone augmentation done after 8 weeks post-extraction.

It is crucial for VRA to achieve primary closure to obtain predictable results. Therefore, the soft tissue quantity and quality play an important role in wound closure. The size of the extraction socket and the pre-extraction conditions determine the time required for the complete healing of the soft tissue maturation. The present study indicated that the after 12-week group demonstrated better vertical ridge augmentation. It can be suspected that the regeneration potential from the extraction sockets may be sustained for up to 16 weeks or more, and the soft tissue volume plays a pivotal role in assisting early bone augmentation.

One of the challenges of VRA is the ability to maintain the space, referred to as space-making. The conventional approach utilizes non-resorbable membranes with a metal framework to provide space-making, such as titanium-reinforced PTFE membranes,^10^, ^11^, ^12^, ^13^, ^14^, ^15^ and titanium mesh.^27^, ^28^, ^29^, ^30^, ^31^, ^32^, ^33^, ^34^ On the contrary, resorbable membranes are not built-in with any reinforced frameworks. Therefore, certain adjunctive devices, such as tenting screws,^26^^,^^35^^,^^36^ and osteosynthesis plates,^37^^,^^38^ can be used. The present study used resorbable to achieve VRA with or without tenting screws. The data indicated that the sites grafted with the combination of resorbable membranes and tenting screws yielded a trend of better vertical bone gain of 4.8 ± 1.74 mm than 4.52± 1.8 mm of the sites without tenting. Still, both showed similar capability to reach the EVBG. It can be speculated that the application of tenting screws in cooperation with resorbable membranes may enhance bone augmentation in early bone augmentation protocol.

The defect dimension is essential for predicting vertical ridge augmentation and maintaining the space. In the present study, both the vertical bone gain (4.7 ± 1.7 mm versus 4.56 ± 1.97) and the chance of vertical bone gain exceeding EVBG (89.66 % versus 66.67 %) were superior for the single space sites than the multiple teeth sites, given that the single space sites exhibit better space making property than the multiple ones. In addition to the healing potential, the barrier function of exclusive ability in time contributes to bone regeneration and maturation.^10^ For the resorbable membrane, a trend for better vertical bone gain favors long-lasting products such as cross-linked collagen membranes.^39^^,^^40^

The early bone augmentation protocol achieved comparable vertical bone gain with minimal complications. 80 % of the time, the vertical bone augmentation exceeded the anticipated level (EVBG), and the average vertical bone gain was 4.64 mm with a maximum true vertical bone gain of 8.57 mm.

## Declaration of competing interest

The authors reported no conflict of interest.
